# The levels of the NMDA receptor co-agonist D-serine are reduced in the substantia nigra of MPTP-lesioned macaques and in the cerebrospinal fluid of Parkinson’s disease patients

**DOI:** 10.1038/s41598-019-45419-1

**Published:** 2019-06-20

**Authors:** Tommaso Nuzzo, Daniela Punzo, Paola Devoto, Elena Rosini, Silvia Paciotti, Silvia Sacchi, Qin Li, Marie-Laure Thiolat, Celine Véga, Massimo Carella, Manolo Carta, Fabrizio Gardoni, Paolo Calabresi, Loredano Pollegioni, Erwan Bezard, Lucilla Parnetti, Francesco Errico, Alessandro Usiello

**Affiliations:** 1 Translational Neuroscience Unit, IRCCS Casa Sollievo della Sofferenza, 71013 San Giovanni Rotondo, Italy; 2 Laboratory of Behavioural Neuroscience, Ceinge Biotecnologie Avanzate, 80145 Naples, Italy; 3 Department of Environmental, Biological and Pharmaceutical Science and Technologies, Università degli Studi della Campania “Luigi Vanvitelli”, 81100 Caserta, Italy; 4 Department of Biomedical Sciences, University of Cagliari, 09042 Monserrato, Italy; 5 Department of Biotechnology and Life Sciences, Università degli Studi dell’Insubria, 21100 Varese, Italy; 6 Department of Pharmaceutical Sciences, University of Perugia, 06122 Perugia, Italy; 7 Motac Neuroscience, UK-M15 6WE, Manchester, United Kingdom; 8 Institute of Lab Animal Sciences, China Academy of Medical Sciences, Beijing, China; 9 Université de Bordeaux, Institut des Maladies Neurodégénératives, Bordeaux, France; 10 Centre National de la Recherche Scientifique Unité Mixte de Recherche 5293, Institut des Maladies Neurodégénératives, Bordeaux, France; 11 Dipartimento di Scienze Farmacologiche e Biomolecolari (DiSFeB), Università degli Studi di Milano “La Statale”, 20133 Milan, Italy; 12 Department of Medicine, Neurology Clinic, University Hospital of Perugia, 06129 Perugia, Italy; 13 Department of Agricultural Sciences, University of Naples “Federico II”, 80055 Portici, Italy

**Keywords:** Parkinson's disease, Neuroscience

## Abstract

Dysfunction of NMDA receptor (NMDAR)-mediated transmission is supposed to contribute to the motor and non-motor symptoms of Parkinson’s Disease (PD), and to L-DOPA-induced dyskinesia. Besides the main agonist L-glutamate, two other amino acids in the atypical D-configuration, D-serine and D-aspartate, activate NMDARs. In the present work, we investigated the effect of dopamine depletion on D-amino acids metabolism in the brain of MPTP-lesioned *Macaca mulatta*, and in the serum and cerebrospinal fluid of PD patients. We found that MPTP treatment increases D-aspartate and D-serine in the monkey putamen while L-DOPA rescues both D-amino acids levels. Conversely, dopaminergic denervation is associated with selective D-serine reduction in the *substantia nigra*. Such decrease suggests that the beneficial effect of D-serine adjuvant therapy previously reported in PD patients may derive from the normalization of endogenous D-serine levels and consequent improvement of nigrostriatal hypoglutamatergic transmission at glycine binding site. We also found reduced D-serine concentration in the cerebrospinal fluid of L-DOPA-free PD patients. These results further confirm the existence of deep interaction between dopaminergic and glutamatergic neurotransmission in PD and disclose a possible direct influence of D-amino acids variations in the changes of NMDAR transmission occurring under dopamine denervation and L-DOPA therapy.

## Introduction

Parkinson’s Disease (PD) is a chronic neurological disorder characterized by the degeneration of the dopaminergic nigrostriatal pathway, which results in progressive motor dysfunction associated to non-motor symptoms, including apathy and dementia^1,2^. Pharmacological approaches to PD predominantly target the dopaminergic system, and dopamine (DA) replacement by its precursor L-3,4-dihydroxyphenylalanine (L-DOPA) remains the gold-standard treatment^3^. However, chronic L-DOPA exposure leads to motor side effects, including wearing-off and L-DOPA-induced dyskinesia^4^.

Progressive degeneration of the midbrain dopaminergic neurons results in an imbalance within cortico-basal ganglia circuit^5^ and is associated with altered glutamatergic transmission in both preclinical models and PD patients. There is indeed consistent agreement about the implication of dysfunctions of glutamatergic system in basal ganglia in PD pathophysiology, as well as in the motor disturbances associated with L-DOPA therapy^2,6–11^. Furthermore, the occurrence of altered stimulation of NMDA-type ionotropic glutamate receptors (NMDARs) is hypothesized to contribute to the molecular events underpinning excitation-mediated neuronal damage and apoptosis in PD brain^12,13^.

In the mammalian brain, besides the main excitatory amino acid L-glutamate (L-Glu), two amino acids in D configuration, D-serine (D-Ser) and D-aspartate (D-Asp), are known to influence NMDAR-mediated transmission. In particular, D-Ser is known to stimulate the glycine-binding site of NMDARs^14,15^, while D-Asp binds to the glutamate site of this receptor subclass^16^. Conversely, their respective L-enantiomers, L-Ser and L-Asp, serve mainly as building blocks of proteins and metabolic intermediates^17,18^. In addition, L-Asp has long been recognized as a selective agonist for NMDARs, although its role as a neurotransmitter is still debated^19^. D-Ser is generated from L-Ser by the pyridoxal phosphate (PLP)-dependent enzyme serine racemase (SR)^20,21^, and its degradation occurs through an oxidative deamination catalyzed by D-amino acid oxidase (DAAO)^22,23^. Conversely, the precise mechanism underlying the endogenous production of D-Asp is not yet understood, although it is well established that its degradation is catalyzed by D-aspartate oxidase (DDO) enzyme^16,24^. So far, only a few investigations have addressed the involvement of these D-amino acids in the dysfunctional glutamate transmission found in PD. In this regard, preclinical observations showed altered D-Ser concentrations in the brain of MPTP- and 6-OHDA-lesioned rodents^25–27^. A beneficial effect of D-Ser supplementation on motor and behavioural symptoms of PD patients treated with L-DOPA has also been documented in a clinical trial^28^. Interestingly, this is in line with the therapeutic benefit displayed by another NMDAR enhancer, sarcosine (a type 1 glycine transporter inhibitor), on neuropsychiatric symptoms of PD^29^. On the other hand, observations in *Ddo* knockout (*Ddo*^−/−^) mice indicated that abnormally high D-Asp levels trigger age-dependent neuroinflammation and cell death in midbrain dopaminergic neurons^30^, as well as a precocious onset of L-DOPA-induced dyskinesia^31^.

In this work, by using a well-validated MPTP-lesioned primate model of PD, we first explored the consequences of DA denervation and L-DOPA therapy upon D-Ser and D-Asp metabolism in the putamen, substantia nigra (SN) and medial frontal gyrus (MFG) of *Macaca mulatta*. Then, in order to assess the translational relevance of preclinical studies in parkinsonian monkeys, we analyzed the concentration of these NMDAR-related modulators in the serum and cerebrospinal fluid (CSF) of PD patients.

## Results

### MPTP treatment in parkinsonian monkeys induces striatal increase in D-aspartate levels, which is normalized by L-DOPA therapy

In this study, we used MPTP-lesioned monkeys because, differently to the commonly used 6-OHDA and MPTP-treated rodents, this primate PD model allows to better approximate the real pathological situation of PD patients^32,33^. Moreover, a subgroup of parkinsonian monkeys was chronically treated with L-DOPA in order to elicit dyskinetic motor disturbances and, thus, approximate the complications of L-DOPA treatment in patients.

**Figure 1:**
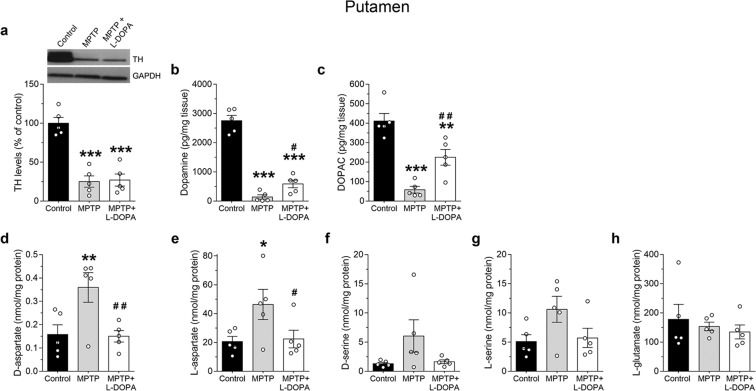
Effect of MPTP-induced striatal dopamine depletion and L-DOPA supplementation on free amino acids levels in the monkey putamen. (**a**) TH protein expression was detected by Western blotting in untreated (control), MPTP- and (MPTP + L-DOPA)-treated monkeys (n = 5 monkeys/treatment). TH variations are expressed as percentage of the control group. Representative blot of TH immunodensity comparing the experimental groups are shown above the graph. GAPDH was used to normalize for variations in loading and transfer. (**b**) Dopamine and **(c)** DOPAC (expressed as pg/mg of fresh tissue), and free amino acids (**d**) D-aspartate, (**e**) L-aspartate **(f)** D-serine, **(g)** L-serine and **(h)** L-glutamate (expressed as nmol/mg protein) were measured by HPLC in the same samples used for TH detection. All free amino acids were detected in a single run. **p < 0.01, ***p < 0.0001, compared to control group; ^#^*p* < 0.05, ^##^*p* < 0.01, compared to MPTP-treated group (Fisher’s *post-hoc*). Dots represent the single subjects’ values while bars illustrate the means ± SEM.

We first analyzed the content of DA and its metabolite, 3,4-Dihydroxyphenylacetic acid (DOPAC), in the putamen of *Macaca mulatta* treated with MPTP or MPTP + L-DOPA. MPTP treatment induced nigro-striatal dopaminergic degeneration in monkeys, as indicated by dramatic decrease in striatal tyrosine hydroxylase (TH) expression (~75%), in both MPTP and MPTP + L-DOPA groups, when compared to controls (one-way ANOVA, F_(2,12)_ = 32.62, *p* < 0.0001; Ctrl *vs* MPTP, *p* < 0.0001, Ctrl *vs* MPTP + L-DOPA, *p* < 0.0001, Fisher’s *post-hoc* comparison; Fig. 1a). In agreement with dopaminergic neuronal degeneration, HPLC analysis showed a significant effect of MPTP treatment on the levels of DA and its metabolite DOPAC (DA: F_(2,12)_ = 105.2, *P* < 0.0001; DOPAC: F_(2,12)_ = 27.03, *P* < 0.0001; Fig. 1b,c). In particular, we observed a severe depletion of both molecules in parkinsonian monkeys (Ctrl *vs* MPTP, mean ± SEM of pg/mg tissue; DA: 2754.0 ± 182.1 *vs* 141.0 ± 73.3, *p* < 0.0001; DOPAC: 411.0 ± 39.1 *vs* 58.2 ± 16.9, *p* < 0.0001; Fig. 1b,c). DA and DOPAC levels were still significantly reduced compared to control group, also after L-DOPA administration (Ctrl *vs* MPTP + L-DOPA, mean ± SEM of pg/mg tissue; DA: 2754.0 ± 182.1 *vs* 586.8 ± 131.2, *p* < 0.0001; DOPAC: 411.0 ± 39.1 *vs* 224.6 ± 40.5, *p* = 0.0022; Fig. 1b,c) as previously reported^34,35^. However, L-DOPA partially counteracted this reduction, as shown by significantly increased DA and DOPAC content in monkeys treated with MPTP + L-DOPA, compared to MPTP-lesioned animals (MPTP *vs* MPTP + L-DOPA, mean ± SEM of pg/mg tissue; DA: 141.0 ± 73.3 *vs* 586.8 ± 131.2, *p* = 0.0393; DOPAC: 58.2 ± 16.9 *vs* 224.6 ± 40.5, *p* = 0.0047; Fig. 1b,c) as previously reported^34,35^.

Then, we performed HPLC analyses in order to determine the striatal concentrations of amino acids involved in NMDAR modulation in MPTP-treated monkeys with or without L-DOPA administration, compared to naïve controls. Our data showed a significant main effect of MPTP treatment on striatal D-Asp levels (F_(2,12)_ = 6.64, *p* = 0.0115; Fig. 1d). Notably, MPTP was able to increase D-Asp levels in treated macaques (Ctrl vs MPTP, mean ± SEM of nmol/mg protein; 0.16 ± 0.04 *vs* 0.36 ± 0.06, *p* = 0.0092; Fig. 1d), and such increase was normalized by chronic L-DOPA administration (Ctrl *vs* MPTP + L-DOPA, mean ± SEM of nmol/mg protein; 0.16 ± 0.04 *vs* 0.15 ± 0.02, *p* = 0.9115; MPTP *vs* MPTP + L-DOPA, 0.36 ± 0.06 *vs* 0.15 ± 0.02, *p = *0.0075; Fig. 1d). MPTP also caused a significant variation in the striatal L-Asp content (F_(2,12)_ = 3.887, *p* = 0.0500). Further statistical analysis revealed that MPTP alone was able to increase L-Asp levels (Ctrl *vs* MPTP, mean ± SEM of nmol/mg proteins; 20.59 ± 3.60 *vs* 46.33 ± 10.45, *p* = 0.0282; Fig. 1e), which were normalized by L-DOPA treatment (Ctrl *vs* MPTP + L-DOPA, mean ± SEM of nmol/mg proteins; 20.59 ± 3.60 *vs* 22.34 ± 6.12, *p* = 0.8681; MPTP *vs* MPTP + L-DOPA, 46.07 ± 10.64 *vs* 22.34 ± 6.12, *p* = 0.0423; Fig. 1e). As a consequence of the concomitant variations in D- and L-Asp, we found that the ratio between D-Asp and total Asp (D- + L-Asp) was unchanged among the three groups analyzed (one-way ANOVA, F_(2,12)_ = 0.49, *p* = 0.6236).

Interestingly, an increase, although not significant, was observed also in both D- and L-Ser levels in parkinsonian monkeys, which was again rescued by L-DOPA therapy (D-Ser: F_(2, 12)_ = 2.67, *p* = 0.1101; L-Ser: F_(2,12)_ = 3.05, *p* = 0.0849; Fig. 1f,g). These results are in line with those obtained in rat and mice, which showed a significant increase of striatal D-Ser levels as a consequence of bilateral DA denervation induced either by 6-OHDA or MPTP treatment, respectively^25,27^. The tendency to the transient increase of both D- and L-Ser in MPTP-treated monkeys was reflected in unchanged D-Ser/total Ser ratio among the three groups analyzed (one-way ANOVA, F_(2,12)_ = 1.51, *p* = 0.2592). Overall, the ability of DA depletion and partial DA supplementation by L-DOPA to respectively increase and normalize the levels of D-Asp and D-Ser, and their relevant L-enantiomers, highlight the occurrence of a functional interaction between striato-nigral DA levels and amino acids homeostasis in the putamen of parkinsonian monkeys.

Finally, we measured the striatal content of L-Glu. Notably, HPLC measurements revealed no detectable differences among the experimental groups (F_(2,12)_ = 0.41, *p* = 0.6733; Fig. 1h).

### MPTP treatment induces the selective reduction of the NMDAR co-agonist, D-serine, in the substantia nigra of parkinsonian monkeys

**Figure 2:**
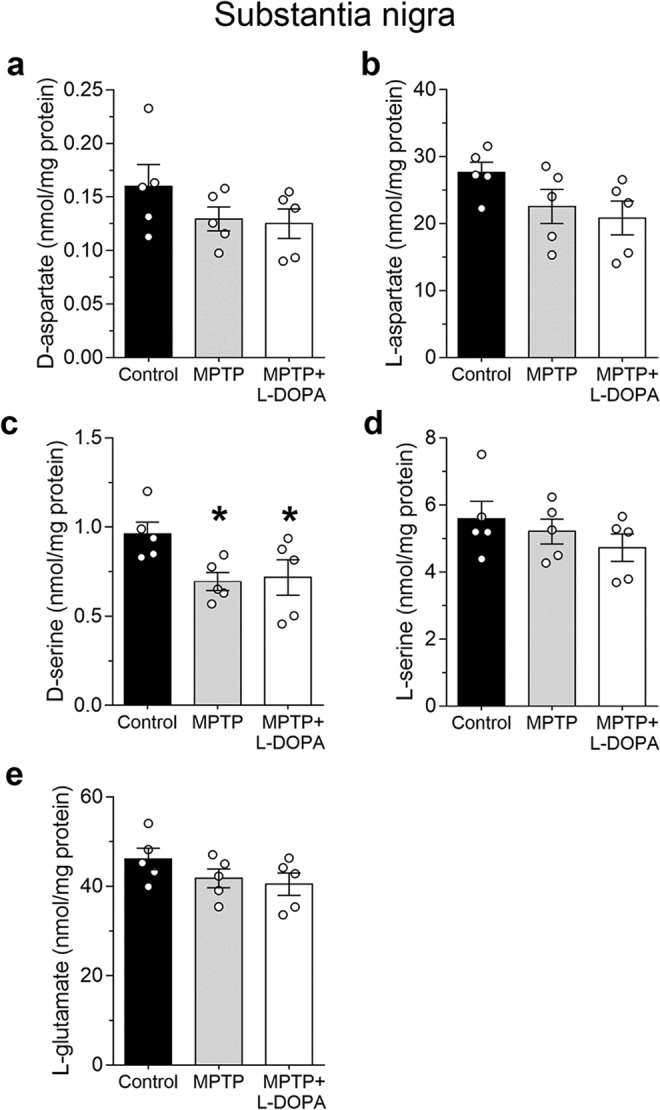
Effect of MPTP-induced striatal dopamine depletion and L-DOPA supplementation on free amino acids levels in the monkey *substantia nigra*. Free amino acids (**a**) D-aspartate, (**b**) L-aspartate **(c)** D-serine, **(d)** L-serine and **(e)** L-glutamate (expressed as nmol/mg protein) were measured by HPLC in untreated (control), MPTP- and (MPTP + L-DOPA)-treated monkeys (n = 5 monkeys/treatment). All free amino acids were detected in a single run. *p < 0.01, compared to control group (Fisher’s *post-hoc*). Dots represent the single subjects’ values while bars illustrate the means ± SEM.

The changes in amino acids levels found in monkey putamen led us to evaluate their content also in a brain region functionally linked to the putamen and critically involved in PD, such as the SN. HPLC analysis showed no significant changes in D- and L-Asp levels in animals treated with MPTP or MPTP + L-DOPA, compared to controls (one-way ANOVA: D-Asp: F_(2,12)_ = 1.47, *p* = 0.2688; L-Asp: F_(2,12)_ = 2.41, *p* = 0.1315; Fig. 2a,b), as well as in L-Glu levels among the different experimental groups analyzed (F_(2,12)_ = 1.61, *p* = 0.2407; Fig. 2e). Conversely, statistical analysis indicated that MPTP significantly affected D-Ser levels (F_(2,12)_ = 3.91, *p* = 0.0493; Fig. 2c). In particular, we observed a decrease in D-Ser content (~30%) in monkeys treated with MPTP alone and with MPTP + L-DOPA, compared to untreated animals (Ctrl *vs* MPTP, mean ± SEM of nmol/mg proteins; 0.96 ± 0.07 *vs* 0.69 ± 0.05, *p* = 0.0268; Ctrl vs MPTP + L-DOPA, 0.96 ± 0.07 *vs* 0.72 ± 0.10, *p* = 0.0398; Fisher’s *post-hoc* comparisons; Fig. 2c). On the other hand, no significant changes in the levels of L-Ser were detected in MPTP-treated macaques, with or without L-DOPA therapy (F_(2,12)_ = 0.96, *p* = 0.4096; Fig. 2d). The analysis of D-Ser/total Ser ratio revealed significant changes among control, MPTP- and MPTP + L-DOPA-treated monkeys (one-way ANOVA: F_(2,12)_ = 5.12, *p* = 0.0247). In particular, in line with D-Ser variation, this ratio was significantly reduced in MPTP-treated monkeys, compared to control animals (Ctrl *vs* MPTP, mean ± SEM of %; 16.83 ± 0.95 *vs* 12.33 ± 1.40, *p = *0.0082). On the other hand, as a consequence of a slight L-Ser decrease in MPTP + L-DOPA-treated monkeys, D-Ser/total Ser ratio in these animals was comparable to that found in control or MPTP-treated monkeys (Ctrl *vs* MPTP + L-DOPA, mean ± SEM of %, 16.8 ± 0.95 *vs* 15.22 ± 0.50, *p = *0.2788; MPTP *vs* MPTP + L-DOPA, 12.33 ± 1.40 *vs* 15.22 ± 0.50; *p = *0.0657).

Altogether, these results highlight that in the SN of monkeys, MPTP treatment is able to trigger the selective reduction of D-Ser, thus suggesting an involvement of this NMDAR co-agonist in the neurochemical modifications of glutamatergic system associated to PD pathophysiology.

### Unaltered D-aspartate and D-serine content in the medial frontal gyrus of MPTP-treated monkeys

**Figure 3:**
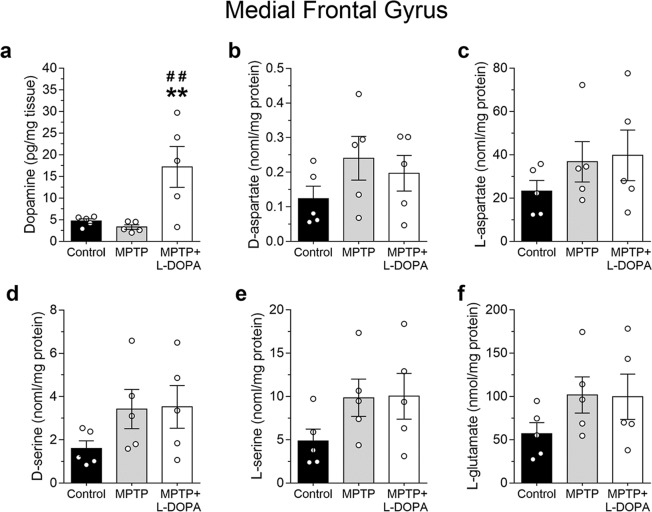
Effect of MPTP-induced striatal dopamine depletion and L-DOPA supplementation on free amino acids levels in the monkey medial frontal gyrus. (**a**) Dopamine (expressed as pg/mg of fresh tissue), and free amino acids (**b**) D-aspartate, (**c**) L-aspartate **(d)** D-serine, **(e)** L-serine and **(f)** L-glutamate (expressed as nmol/mg protein) were measured by HPLC in untreated (control), MPTP- and (MPTP + L-DOPA)-treated monkeys (n = 5 monkeys/treatment). All free amino acids were detected in a single run. **p < 0.01, compared to control group; ^##^*p* < 0.01, compared to MPTP-treated group (Fisher’s *post-hoc*). Dots represent the single subjects’ values while bars illustrate the means ± SEM.

We then addressed DA, DOPAC and amino acids detection in a region of the prefrontal cortex like the MFG. HPLC analysis indicated that MPTP treatment alone did not affect DA levels (one-way ANOVA: F_(2,12)_ = 7.73, *p* = 0.0070; Ctrl *vs* MPTP, mean ± SEM of pg/mg tissue; 4.70 ± 0.52 *vs* 3.30 ± 0.53, *p* = 0.7254; Fisher’s *post-hoc* comparison; Fig. 3a), while chronic L-DOPA administration induced an increase of DA in MPTP-treated animals, compared to vehicle and MPTP alone administration (Ctrl *vs* MPTP + L-DOPA, mean ± SEM of pg/mg tissue; 4.70 ± 0.51 *vs* 17.20 ± 4.71, *p* = 0.0075; MPTP *vs* MPTP + L-DOPA, 3.30 ± 0.52 *vs* 17.20 ± 4.71, *p* = 0.0039; Fig. 3a).

Then, we measured the levels of D- and L-amino acids. One-way ANOVA showed no main effect of MPTP treatment on D- and L-Asp amount (D-Asp: F_(2,12)_ = 1.32, *p* = 0.3044; L-Asp: F_(2,12)_ = 0.94, *p* = 0.4178; Fig. 3b,c). Likewise, D-Ser and L-Ser content was comparable among the different experimental groups (D-Ser: F_(2,12)_ = 1.84, *p* = 0.2014; L-Ser: F_(2,12)_ = 1.91, *p* = 0.1905; Fig. 3d,e). Interestingly, our results are consistent with unaltered D-Ser levels observed in the *post-mortem* cortex of PD brain^36^ and in the cortex of MPTP-lesioned mice^27^. Similarly to Asp and Ser enantiomers, no significant difference in L-Glu content among the different experimental groups analyzed were observed (F_(2,12)_ = 1.47, *p* = 0.2674; Fig. 3f).

Overall, unaltered amino acids levels in the MFG suggest that MPTP is unable to affect their metabolism in a brain region functionally unrelated to basal ganglia circuit^37^, even under L-DOPA-dependent DA increase.

### MPTP treatment induces an increase in *D-amino acid oxidase* mRNA and protein levels in the substantia nigra of monkeys

**Figure 4:**
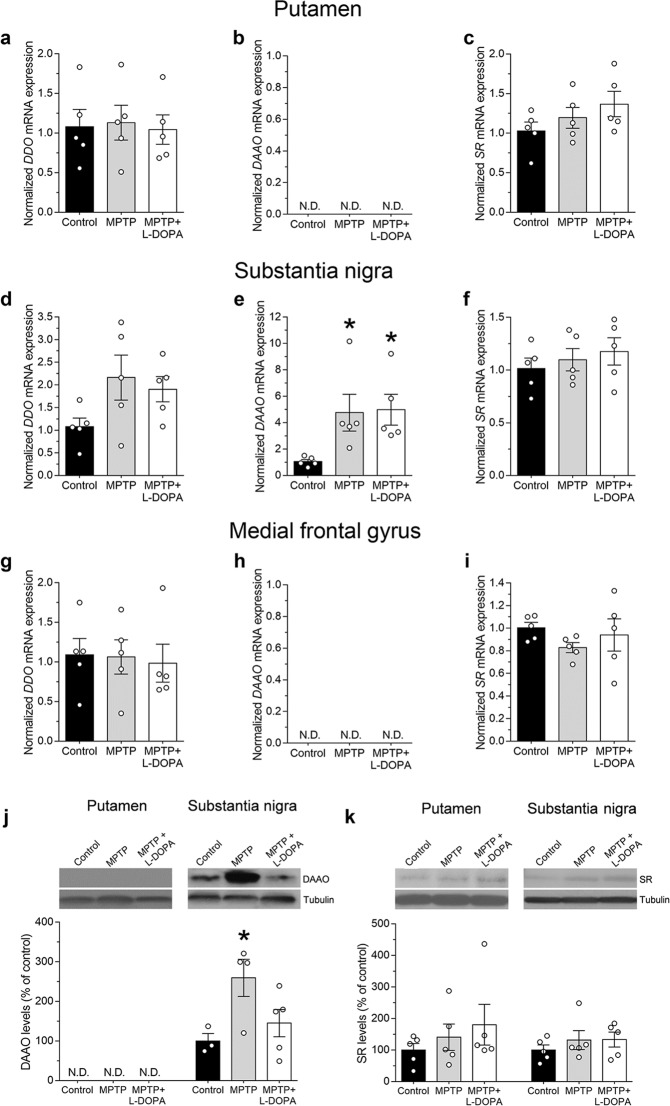
mRNA and protein expression of *DDO, DAAO* and *SR* in the brain of parkinsonian monkeys. **(a,d,g)** *DDO*, **(b,e,h)** *DAAO* and **(c,f,i)** *SR* mRNA expression was detected by quantitative RT-PCR in the **(a–c)** putamen, **(d–f)** *substantia nigra* and **(g-i)** medial frontal gyrus of untreated (control), MPTP- and (MPTP + L-DOPA)-treated monkeys (n = 5 monkeys/treatment). mRNA expression is normalized to the mean of three housekeeping genes and expressed as arbitrary units. **(b,h)** N.D. indicates that *DAAO* transcript was not detectable up to 40 cycles. **(j)** DAAO and **(k)** SR protein levels were detected by Western blotting in the same putamen and *substantia nigra* samples used for quantitative RT-PCR. **(j)** N.D. indicates that DAAO protein was not detectable. Proteins variations are expressed as percentage of the control group. Representative blots of DAAO and SR immunodensity comparing the experimental groups are shown above the graph. Full-length blots are presented in Supplementary Fig. S1. Tubulin was used to normalize for variations in loading and transfer. *p < 0.05, compared to control group (Fisher’s *post-hoc*). Dots represent the single subjects’ values while bars illustrate the means ± SEM.

In order to gain insights into the molecular mechanisms responsible for the D-amino acids variations observed in the putamen and SN of MPTP-treated monkeys, we analyzed the expression of the genes regulating D-Asp (*DDO*) and D-Ser (*DAAO* and *SR*) metabolism. To this aim, we performed quantitative Real-Time PCR (qRT-PCR) analysis in the same brain samples used for HPLC detection. We found no alterations in *DDO* transcript levels within the putamen (one-way ANOVA, F_(2,12)_ = 0.04, *p* = 0.9563; Fig. 4a), SN (F_(2,12)_ = 2.66, *p* = 0.1105; Fig. 4d) and MFG (F_(2,12)_ = 0.06, *p* = 0.9413; Fig. 4g) of parkinsonian monkeys with or without L-DOPA treatment, compared to naïve controls.

We then analysed the expression of the genes regulating D-Ser metabolism. Quantitative RT-PCR revealed that *DAAO* mRNA was undetectable (up to 40 cycles) in the striatal and cortical samples tested (Fig. 4b,h). Conversely, in line with previous evidence in humans^38^, *DAAO* transcript was expressed in the SN (at ∼30 cycles; Fig. 4e) where it is significantly affected by MPTP treatment (F_(2,12)_ = 4.39, *p* = 0.0370; Fig. 4e). In particular, regardless of L-DOPA administration, we observed a significant increase in *DAAO* mRNA levels in parkinsonian monkeys, compared to naïve controls (Ctrl *vs* MPTP: *p* = 0.0285; Ctrl *vs* MPTP + L-DOPA: *p* = 0.0216, Fisher’s *post-hoc* comparisons; Fig. 4e). On the other hand, qRT-PCR data revealed no differences in *SR* mRNA expression among the experimental groups in each brain region analyzed (putamen: F_(2,12)_ = 1.55, *p* = 0.2526; SN: F_(2,12)_ = 0.52, *p* = 0.6092; MFG: F_(2,12)_ = 0.97, *p* = 0.4060; Fig. 4c,f,i).

In order to understand whether gene transcription results are reflected also at translational level and whether changes in D-amino acids content could be functionally explained by variations in protein expression, we investigated DAAO and SR protein content in the brain of MPTP-treated macaques, compared to naïve animals. In line with mRNA detection, Western blot analysis indicated that DAAO protein was undetectable in the putamen samples (Fig. 4j). Conversely, we found a significant main effect of MPTP on DAAO protein levels in the SN (F_(2,9)_ = 4.28, *p* = 0.0495; Fig. 4j). In particular, in line with qRT-PCR analysis, MPTP treatment was associated to a significant increase in DAAO protein in parkinsonian monkeys (Ctrl *vs* MPTP: *p* = 0.0230; Fig. 4j). However, chronic L-DOPA supplementation was able to normalize the expression of this enzyme (Ctrl *vs* MPTP + L-DOPA: *p* = 0.4396; Fig. 4j). Finally, no significant changes in SR levels in both putamen and SN samples were detected among different experimental groups (putamen: F_(2,12)_ = 0.76, *p* = 0.4884; SN: F_(2,12)_ = 0.60, *p* = 0.5608; Fig. 4k).

### Analysis of glutamatergic NMDA, AMPA, mGLU receptors levels in the putamen and substantia nigra of MPTP-treated parkinsonian monkeys

Based on dysfunctional glutamatergic transmission observed in preclinical models and PD patients^13^, we measured the total protein amounts of the main NMDA and AMPA receptor subunits, and of the metabotropic glutamate receptors, mGluR2/3 and mGluR5, in the putamen and SN of MPTP-treated macaques with and without L-DOPA treatment, compared to naïve animals.

**Figure 5:**
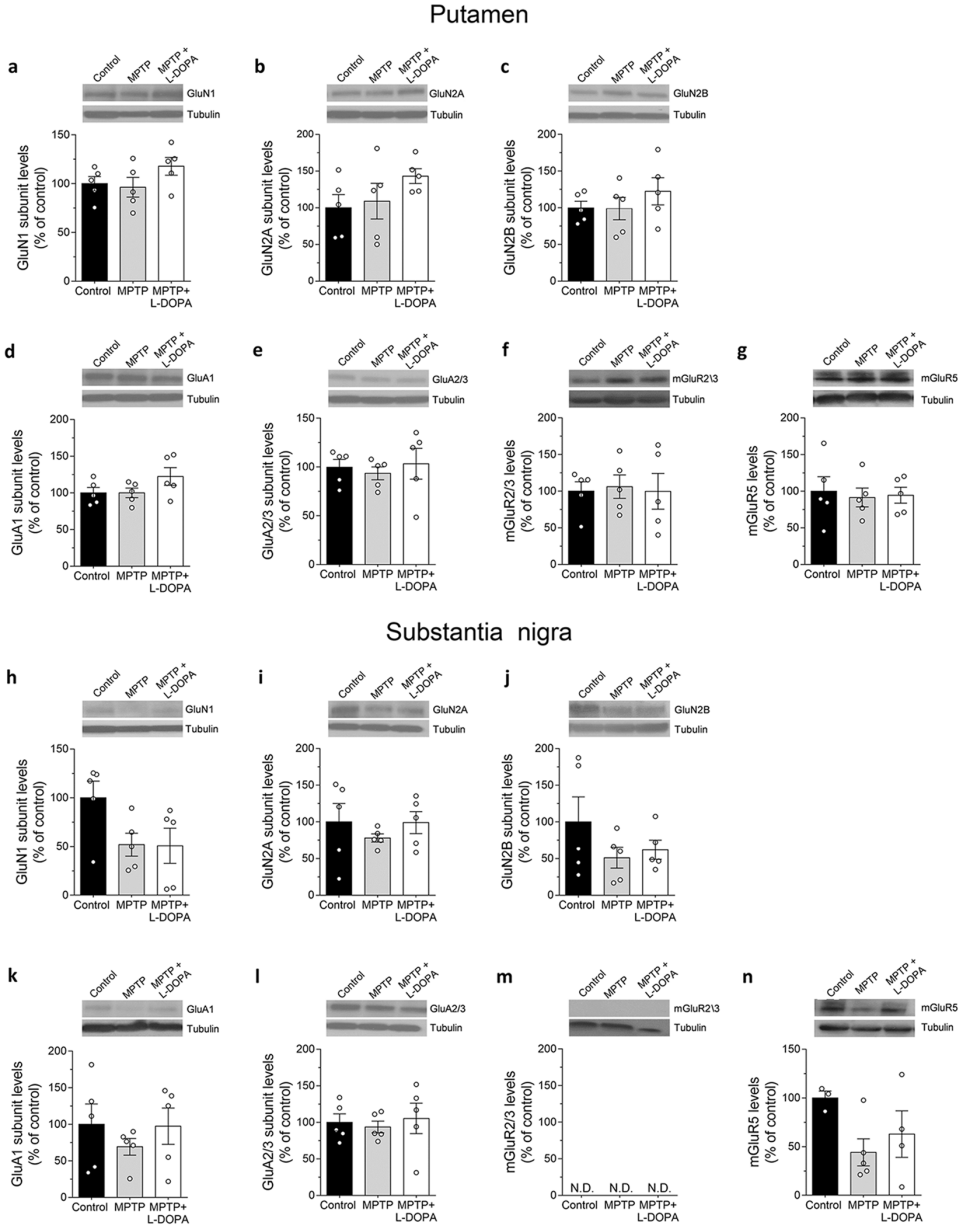
Expression levels of glutamatergic receptors in the brain of parkinsonian monkeys. Protein expression levels of the **(a–c, h–j)** NMDARs subunits **(a,h)** GluN1, **(b,i)** GluN2A, **(c,j)** GluN2B, **(d–e, k–l)** AMPAR subunits **(d,k)** GluA1 and **(e,l)** GluA2/3, and **(f,g, m,n)** metabotropic glutamate receptors **(f,m)** mGluR2/3 and **(g,n)** mGluR5 were detected in the **(a–g)** putamen and **(h–n)** substantia nigra of untreated (control), MPTP- and (MPTP + L-DOPA)-treated monkeys (n = 5 monkeys/treatment)by Western blotting. **(m)** N.D. indicates that protein levels were not detectable. Proteins variations are expressed as percentage of the control group. Representative blots of each subunit or receptor immunodensity comparing the experimental groups are shown above the graph. Tubulin was used to normalize for variations in loading and transfer. Dots represent the single subjects’ values while bars illustrate the means ± SEM.

Western blot analysis indicated no main differences in the striatal expression of the NMDAR subunits GluN1, GluN2A and GluN2B between parkinsonian monkeys and controls (one-way ANOVA, GluN1: F_(2,12)_ = 1.67, *p* = 0.2294; GluN2A: F_(2,12)_ = 1.53, *p* = 0.2549; GluN2B: F_(2,12)_ = 0.81, *p* = 0.4685; Fig. 5a-c). Similarly, MPTP treatment did not perturb the expression of the AMPAR subunits GluA1 and GluA2/3 (GluA1: F_(2,12)_ = 2.10, *p* = 0.1689; GluA2/3: F_(2,12)_ = 0.21, *p* = 0.8106; Fig. 5d,e), and metabotropic glutamate receptors mGluR2/3 and mGluR5 (mGluR2/3: F_(2,12)_ = 0.04, *p* = 0.9613; mGluR5: F_(2,12)_ = 0.08, *p* = 0.9202; Fig. 5f,g). In agreement with our results, previous reports did not observe significant changes in striatal glutamate receptor levels in the non-human primate model both by binding studies^39,40^ and by evaluation of total protein levels through Western blotting^10,41^. Unlike, fractioning experiments showed altered expression of AMPA and NMDAR subunits in MPTP-treated macaques^10,42^, thus suggesting that altered synaptic localization of specific glutamate receptor subtypes and consequent functional alteration, rather than an aberrant total expression level, represent the main event at striatal excitatory synapses in PD^4,7,8^.

Differently from the putamen, MPTP treatment induced a L-DOPA-insensitive trend to reduction of the fundamental GluN1 subunit in the SN of parkinsonian monkeys (F_(2,12)_ = 3.13, *p* = 0.0802; Fig. 5h). Conversely, the levels of GluN2A subunit appeared comparable among the different experimental groups (F_(2,12)_ = 0.53, *p* = 0.6032; Fig. 5i). Similarly to GluN1, also the GluN2B expression was reduced in the SN of parkinsonian monkeys, although such decrease failed to reach the statistical significance (F_(2,12)_ = 1.30, *p* = 0.3083; Fig. 5j). Western blot analysis also indicated unaltered levels of the AMPAR subunits, GluA1 and GluA2/3 (GluA1: F_(2,12)_ = 0.57, *p* = 0.5787; GluA2/3: F_(2,12)_ = 0.16, *p* = 0.8517; Fig. 5k,l). Furthermore, consistently with previous studies^43^, we confirmed that mGluR2/3 protein was undetectable in the SN samples (Fig. 5m), while a trend to decrease of mGluR5 levels in MPTP-treated animals was observed, compared to controls (F_(2,9)_ = 2.39, *p* = 0.1473; Fig. 5n).

### D-serine concentration is reduced in the cerebrospinal fluid of L-DOPA-free Parkinson’s disease patients

**Figure 6:**
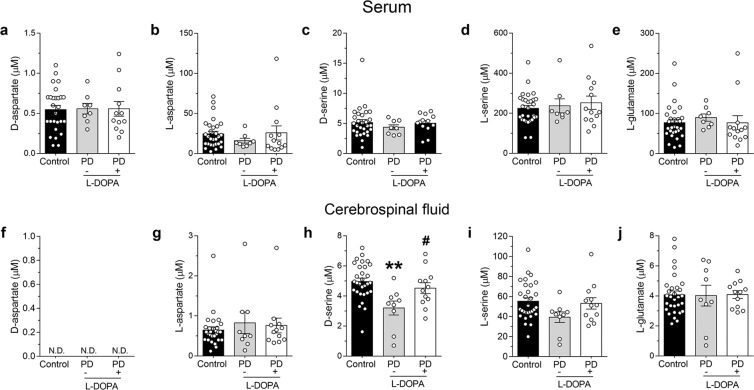
Free amino acids levels in the serum and cerebrospinal fluid of patients with Parkinson’s disease. Free amino acids (**a**,**f**) D-aspartate, (**b,g**) L-aspartate **(c,h)** D-serine, **(d,i)** L-serine and **(e,j)** L-glutamate (expressed as μM) were measured by HPLC **(a-e)** in the serum of control subjects (n = 27 for D-aspartate; n = 29 for other amino acids), L-DOPA-free (n = 8) and L-DOPA-treated (n = 12 for D-aspartate; n = 13 for other amino acids) PD patients, and **(f–j)** in the cerebrospinal fluid of control subjects (n = 28 for L-aspartate; n = 30 for other amino acids), L-DOPA-free (n = 9) and L-DOPA-treated (n = 12) PD patients. **(f)** N.D. indicates that D-aspartate levels were not detectable since they were below the detection limit (<0.01 pmol). In each tissue, all free amino acids were detected in a single run. **p < 0.01, compared to control group; ^#^*p* < 0.05, compared to L-DOPA-free PD patients (Dunn’s test). Dots represent the single subjects’ values while bars illustrate the means ± SEM.

To translate our preclinical observations to humans, we measured the serum content of Asp and Ser enantiomers, and L-Glu in PD patients and control subjects. HPLC analysis revealed unchanged D-Asp content in L-DOPA-free and L-DOPA-treated PD patients, compared to control subjects (Kruskal-Wallis test, *p* = 0.9370; Fig. 6a). Similarly, we revealed no significant L-Asp alterations among experimental groups (*p* = 0.3935; Fig. 6b). We also found comparable amount of D-Ser and L-Ser in the serum of both L-DOPA-free and L-DOPA-treated PD patients, compared to control subjects (D-Ser: *p* = 0.5233; L-Ser: *p* = 0.8616; Fig. 6c,d). Likewise, serum L-Glu content was unaltered among the three groups analyzed (*p* = 0.1221; Fig. 6e).

We extended HPLC analysis to the CSF of the same patients. Notably, we found that D-Asp content was below our HPLC detection limit (<0.01 pmol; Fig. 6f) in all samples analyzed, while the L-Asp content was unaltered in L-DOPA-free and L-DOPA-treated PD patients, compared to control subjects (Kruskal-Wallis test, *p* = 0.9475; Fig. 6g). On the other hand, statistical analysis revealed a significant variation of D-Ser levels among the three groups analyzed (*p* = 0.0043; Fig. 6h). In this regard, the following *post-hoc* comparison showed a significant decrease (~35%) of D-Ser content in the CSF of PD patients without L-DOPA treatment, compared to controls (Ctrl *vs* L-DOPA-free, mean ± SEM of µM, 4.96 ± 0.22 *vs* 3.20 ± 0.46, Dunn’s test, *p* = 0.0010; Fig. 6h). Interestingly, such decrease was not found in PD patients treated with L-DOPA (Ctrl *vs* L-DOPA, mean ± SEM of µM, 4.96 ± 0.22 *vs* 4.52 ± 0.36; *p* = 0.2616; L-DOPA-free *vs* L-DOPA, 3.20 ± 0.46 *vs* 4.52 ± 0.36; *p* = 0.0497; Fig. 6h). Unlike D-Ser, we did not reveal significant alterations in L-Ser content in the CSF of PD subjects, regardless of L-DOPA treatment, compared to control subjects (*p* = 0.1068; Fig. 6i). The variations in D-Ser content among the three groups analyzed were also confirmed by the analysis of D-Ser/total Ser ratio (Ctrl *vs* L-DOPA-free, mean ± SEM of %, 8.57 ± 0.32 *vs* 7.01 ± 0.30; *p* = 0.0117; Ctrl *vs* L-DOPA, 8.57 ± 0.32 *vs* 8.15 ± 0.52; *p* = 0.5458; L-DOPA-free *vs* L-DOPA, 7.01 ± 0.30 *vs* 8.15 ± 0.52; *p* = 0.0884).

Finally, we observed comparable L-Glu content in control, L-DOPA-free and L-DOPA-treated PD patients (*p* = 0.8611; Fig. 6j).

## Discussion

Nowadays, structural, functional and synaptic modifications occurring at NMDARs in PD represent a topic of intense investigation since these receptors are postulated to play a primary role in the progression and treatment of this neurodegenerative disease^13,44–46^. Here, we explored the consequences of dopaminergic denervation on the metabolism of the NMDAR modulators, D-Asp and D-Ser, in the brain of MPTP-lesioned macaques, and in the serum and CSF of PD patients, with or without L-DOPA therapy.

We report for the first time that the gold-standard PD primate model shows a significant D-Ser reduction in the SN. In line with our observation in primates, a reduction of D-Ser content was also reported in the midbrain of MPTP-treated mice^26^. These results suggest that the decreased availability of the NMDAR co-agonist D-Ser may represent a common compensatory mechanism to counteract NMDAR-mediated toxicity and, ultimately, dopaminergic neuronal death in the SN of MPTP models of PD. Moreover, we also found a trend of reduction in the protein expression of the NMDAR subunits, GluN1 and GluN2B, and mGlu5 receptors, thus disclosing the possible existence of an overall hypo-glutamatergic transmission in the SN of parkinsonian monkeys. Interestingly, the neurochemical observations obtained in the SN of MPTP-lesioned monkeys also provide a possible rationale to explain the beneficial effect of D-Ser add-on administration on motor and non-motor symptoms of PD patients^28^. Indeed, D-Ser administration may aid in restoring the endogenous levels of this D-amino acid and, in turn, the balance between NMDAR-related pro-death and pro-survival signaling pathways in the residual midbrain neurons, as hypothesized by Heresco-Levy and coworkers^47^. Yet, amelioration of the defective NMDAR signaling at glycine-binding site in the spared midbrain dopaminergic neurons may enhance nigro-striatal DA release and synaptic transmission^48,49^, thus improving the clinical responses of PD patients. On the other hand, based on the “double-edged sword” of NMDAR stimulation^50,51^, we cannot exclude that long-lasting D-Ser adjuvant treatment in PD patients might determine detrimental effects by increasing the progression-rate of midbrain TH-positive neurons degeneration. Therefore, future investigations are mandatory to confirm D-Ser safety at high doses and, most importantly, for longer treatment periods in large cohorts of PD patients, as previously reported in schizophrenia-diagnosed subjects^52^. Alternatively, to avoid the potential toxicity of D-Ser administration, another possible route might be pursued in PD therapeutics through the inhibition of DAAO by sodium benzoate, which has been already successfully applied in the treatment of schizophrenia and Alzheimer’s disease^53^.

Besides D-Ser decrease in the SN, we observed that MPTP treatment induces a robust augmentation of D-Asp and L-Asp levels in the primate putamen, coupled to a trend increase of D-Ser and L-Ser. Interestingly, the partial restoration of DA levels by L-DOPA was sufficient to rescue the levels of both Asp and Ser enantiomers. Based on the knowledge that NMDAR stimulation enhanced DA synthesis and release within the striatum^48^, and considering the common ability of L-Asp, D-Asp and D-Ser to directly stimulate NMDARs^54–56^, and the role of L-Ser as precursor of D-Ser biosynthesis^21^, we speculate that the variations in these amino acids content under DA denervation represent an attempt of striatal circuitry to counteract the reduction of nigro-striatal dopaminergic transmission. In support of a dynamic interaction between DA levels and neuroactive amino acids, Moratalla and co-workers reported an overall increase in glutamine, glycine and taurine levels in the striatum of unilaterally lesioned 6-OHDA mice, which was normalized by L-DOPA supplementation^57^.

Despite the L-DOPA-sensitive D-Asp alterations reported in the putamen of MPTP-treated monkeys, we found no significant change in *DDO* mRNA expression among the experimental groups. Considering that in the putamen of parkinsonian monkeys both Asp enantiomers were significantly up-regulated, as indicated by the lack of significant difference in the D-Asp/total Asp ratio, we cannot rule out that changes in D-Asp levels may be a secondary effect of L-Asp accumulation and, therefore, independent by direct changes in D-Asp degradation. However, the lack of available selective anti-DDO antibodies prevented to assess whether changes in striatal D-Asp content may also depend on alterations of DDO protein levels. Conversely, the pathway responsible for D-Asp biosynthesis in mammals is still unknown and, therefore, we cannot evaluate potential changes in the *de novo* synthesis of this D-amino acid in parkinsonian monkeys. Nonetheless, recent findings revealed that SR might partially generate D-Asp, in addition to D-Ser^58,59^. However, as discussed below for D-Ser, our results exclude any alteration in both *SR* mRNA and protein levels in MPTP-treated monkeys.

Regarding the metabolic regulation of D-Ser, we did not find any change in *SR* transcript levels between MPTP-lesioned monkeys, with or without L-DOPA administration, and control group, in each brain region analyzed. Differently to what observed by Lu and co-workers in MPTP-treated mice^27^, our experiments indicated also a comparable amount of SR protein among treatment groups in the different brain regions tested, thus suggesting the existence of species-specific SR regulation under PD conditions. On the other hand, we highlighted a significantly increased *DAAO* mRNA expression in the SN of both MPTP and MPTP + L-DOPA groups, coupled to increased DAAO protein levels selectively in MPTP-treated monkeys, thus supporting the reduction in D-Ser found in the SN of these animals. Future studies are required to find out whether the upregulation of *DAAO* takes place in astrocytes and/or in dopaminergic midbrain neurons, where the expression of this gene has been previously detected^60,61^. Interestingly, despite increased *DAAO* mRNA levels, we found that L-DOPA supplementation normalized DAAO protein expression in PD monkeys. Therefore, while our data suggest that the lower content of D-Ser in the SN of MPTP-lesioned monkeys originates from the over-expression of *DAAO* gene, it remains still unclear whether other mechanisms contribute to down-regulate D-Ser levels in L-DOPA-treated PD monkeys. Future studies are warranted to clarify this issue, although we excluded a direct effect of L-DOPA and DA on human DAAO and SR activity in *in vitro* assays (see Supplementary Results and Supplementary Tables).

In addition to D-Ser decrease in the SN of MPTP-lesioned monkeys, in the present work we unveiled a significant D-Ser reduction also in the CSF of L-DOPA-free PD patients. This result suggests that profound changes in NMDAR-mediated neurotransmission occur in PD patients, as well as in animal models of PD, and these alterations involve the modulation of the co-agonist D-Ser, rather than the main agonist L-Glu. Of note, L-DOPA therapy in patients is able to normalize D-Ser content at control levels, further indicating the existence of a functional interaction between DA and D-Ser. However, future studies are mandatory to identify the specific cerebral regions responsible for D-Ser alterations found in the CSF of L-DOPA-free PD patients. This issue gains more importance if we consider that in parkinsonian monkeys, DA depletion and its replacement with L-DOPA affect D-Ser levels in a region-specific manner. Unlike CSF, we found that the serum levels of D-Ser are comparable among the different groups analyzed, implying that the metabolic alteration of this NMDAR co-agonist in PD is selective for the central nervous system, rather than being a more generalized event involving peripheral organs.

Experimental limitations should be taken into account for the interpretation of our data. First, the low number of monkey brain and human serum/CSF samples could affect the robustness of our observations. Second, the subjects used as control group (other neurological disorders) suffer from heterogeneous clinical diseases including headache, epilepsy, psychiatric disorders, and white matter lesions (see Table 1) that may underlie dysfunctions in glutamatergic system. Therefore, we cannot exclude that this potential bias may have masked further amino acids deregulations occurring in the serum and/or CSF of PD patients, thus impacting as confounding factor on our neurochemical analyses. Third, in regard to SN samples, we could not discriminate between *pars compacta* and *pars reticulata*. Thus, the MPTP-dependent changes in D-Ser concentration observed in the SN could represent an underestimation of what we might have found in the *pars compacta* alone, which is the area selectively involved in PD-related cell death. Fourth, the analysis of the whole monkey brain homogenates does not allow us to understand whether changes in D-amino acids levels are due to loss of DA cell bodies or originate from expression changes in other neuronal or glial sources in response to DA neurons loss. Moreover, we cannot dissociate between total content and extracellular active fraction of amino acids. Therefore, while the observations in the CSF most likely reflect the neurochemical content of the extracellular *milieu*, further *in vivo* microdialysis studies are necessary in PD monkey brain to understand whether the variations observed in homogenates mirror those occurring at extracellular level.

**Table 1:** Demographic and clinical characteristics of Parkinson’s disease and control subjects. Abbreviations: F = female, M = male, PD = Parkinson’s Disease.

Control				Parkinson’s disease				
*ID*	*Sex*	*Age (years)*	*Diagnosis*	*ID*	*Sex*	*Age (years)*	*Diagnosis*	*L-DOPA therapy*
1	F	73	Behavior disorders	1	M	64	PD	No
2	M	46	Headache	2	M	63	PD	No
3	M	63	Epilepsy with cognitive deficits	3	F	43	PD	No
4	F	56	Behavior disorders	4	M	?	PD	No
5	F	74	Epilepsy	5	M	64	PD	No
6	F	71	Polyfactorial	6	M	73	PD	No
7	F	53	White matter injury	7	M	69	PD	No
8	M	67	Polyfactorial	8	F	60	PD	No
9	F	64	Subjective memory impairment	9	F	66	PD	No
10	F	68	Control	10	M	86	PD	Yes
11	M	48	Subjective memory impairment	11	M	61	PD	Yes
12	M	72	Metabolic encephalopathy	12	M	43	PD	Yes
13	F	60	Transient global amnesia	13	M	70	PD	Yes
14	F	69	Psychiatric	14	F	74	PD	Yes
15	M	68	Epilepsy	15	M	81	PD	Yes
16	M	78	Wernicke’s encephalopathy	16	F	59	PD	Yes
17	F	51	Vascular encephalopathy	17	M	74	PD	Yes
18	M	76	Cognitive deficits	18	F	61	PD	Yes
19	M	67	Senile psychosis	19	F	76	PD	Yes
20	F	82	Late-onset epilepsy with cognitive deficits	20	M	75	PD	Yes
21	F	49	Epilepsy	21	M	64	PD	Yes
22	M	72	Dysmetabolic polyneuropathy with cognitive deficits	22	M	63	PD	Yes
23	F	77	Other neurological disorder					
24	M	81	III cranial nerve					
25	M	71	Psychiatric with cognitive deficits					
26	F	71	Transient global amnesia					
27	M	64	Transient global amnesia					
28	F	76	Epilepsy					
29	M	65	Psychiatric					
30	F	64	Late-onset epilepsy					
*Total*	*Total*	*Mean ± SEM*		*Total*	*Total*	*Mean ± SEM*		
30	M = 14/F = 16	66.5 ± 1.8		22	M = 15/F = 7	66.1 ± 2.3		

In conclusion, the present study highlights in both non-human primates and humans an involvement of D-amino acids in the pathophysiology of PD and its pharmacological treatment. In particular, we hypothesize that D-Ser and D-Asp variations in the SN and putamen of parkinsonian monkeys might represent adaptive neuronal mechanisms to limit NMDAR-mediated midbrain neurotoxicity and counteract the reduction of nigro-striatal dopaminergic transmission^13,62–65^. On the other hand, changes in the SN of primates provide the first possible explanation for the clinical benefit of D-Ser add-on administration observed in patients with severe PD^28,47^.

## Methods

### Non-human primates

Captive bred female macaques (*Macaca mulatta*, Xierxin, Beijing, PR of China; mean age = 5 ± 1 years; mean weight = 5.3 ± 0.8 kg), were housed in individual primate cages under controlled conditions of humidity (50 ± 5%), temperature (24 ± 1 °C), and light (12 h light/12 h dark cycles, time lights on 8:00 am), and allowing visual contacts and interaction with macaques housed in the adjacent cages. Food and water were available ad libitum and animal care was supervised daily by veterinarians skilled in the healthcare and maintenance of non-human primates. Experiments were carried out in accordance with European Communities Council Directive (2010/63/EU) for care of laboratory animals in an AAALAC-accredited facility following acceptance of study design by the Institute of Lab Animal Science IACUC (Chinese Academy of Medical Sciences, Beijing, China). The tissues used in the present work have been obtained from an experimental brain bank used in several occasions whose experimental conditions are described elsewhere in great details. MPTP-treated non-human primate PD model macaques (n = 10) received daily MPTP hydrochloride injections (0.2 mg/kg, intravenously) until parkinsonian signs appeared^34,66–73^. Once PD motor signs were stable, some of the animals (n = 5) were treated twice daily with an individually titrated dose of L-DOPA that provided maximum reversal of parkinsonian motor signs (Madopar, L-DOPA/carbidopa, 4:1 ratio; range, 9–17 mg/kg). This dose of L-DOPA, defined as 100% dose, was used for chronic L-DOPA treatment, which lasted for 4 to 5 months until dyskinesia stabilized. Animals then received L-DOPA twice a week to maintain a consistent level of dyskinesia before acute drug tests were carried out using a within subject experimental design. At the end of the experiment, all animals were killed by sodium pentobarbital overdose (150 mg/kg, i.v.) 1 h after the last dose of vehicle or L-DOPA (i.e. at peak of antiparkinsonian effect), and the brains were removed quickly after death. Each brain was bisected along the midline and the two hemispheres were immediately frozen by immersion in isopentane (−45 °C) and then stored at −80 °C. Coronal 300 μm-thick sections were cryostat-cut and punches of brain tissue were taken for the following regions: motor striatum (post-commissural dorsal putamen), prefrontal cortex (MFG), and substantia nigra. An average sample size of 6 ± 2 mg was obtained^34,71^.

### Human serum and cerebrospinal fluid collection

Serum and cerebrospinal fluid samples were obtained from the Center for Memory Disturbances, University Hospital of Perugia (Italy). The patients (n = 22 of which 9 were L-DOPA-free and 13 were treated with L-DOPA) were diagnosed with PD according to *United Kingdom Brain Bank Society (UKBBS)* criteria^74,75^. As neurological controls (n = 30), subjects who underwent lumbar puncture or blood sampling for diagnostic reasons but without clinical evidence of dementia were enrolled. The commonest control diagnoses were headache, epilepsy, psychiatric disorders, and white matter lesions. The exclusion criteria for the control group were dementia disorders, atypical parkinsonism (i.e., multiple system atrophy, corticobasal syndrome, progressive supranuclear palsy), and systemic and neoplastic diseases. Groups did not differ significantly for age (Ctrl *vs* L-DOPA-free *vs* L-DOPA-treated, mean ± SEM of years: 66.5 ± 1.8 *vs* 62.7 ± 3.1 *vs* 68.2 ± 3.1, *p = *0.3995, Kruskal-Wallis test) and gender (χ^2^ = 2.396, *p* = 0.3018, χ^2^ test). Further details are reported in Table 1. All subjects included in the study gave their informed written consent to undergo lumbar puncture and to allow us to use the biological samples also for scientific purposes (the sheets of informed written consent report several items explaining thoroughly each issue regarding the meaning and aims of the lumbar puncture and following analysis: what is lumbar puncture; why it is carried out; measurements carried out in biological samples; possible scientific use of them, including their sharing with other centers for scientific purposes). This procedure is routinely done since 2008, according to the Local Ethical Committee approval, CEAS (Comitato Etico Aziende Sanitarie Umbria) (Prot. N. 19369/08/AV, Oct 09 2008). CSF collection was performed according to international guidelines^76^. Briefly, lumbar puncture was performed between 8:00 AM and 10:00 AM, after an overnight fast. CSF (10 mL) was taken from the L3-L4 or L4-L5 interspace, immediately collected into sterile polypropylene tubes (Sarstedt, Code 62.610.201) and gently mixed to avoid possible gradient effects. Within 1 h from collection, CSF sample was centrifuged at 2000 × g for 10 min at room temperature, divided into 0.5 mL aliquots in polypropylene cryotubes (Sarstedt, code 72.730007) and stored at –80 °C. Whole blood was collected by peripheral venipuncture into clot activator tubes (Kima, code 11020) and gently mixed. Sample was stored upright for 30 min at room temperature to allow blood to clot, and centrifuged at 2000 × g for 10 min at room temperature. Serum was aliquoted (0.5 ml) in polypropylene cryotubes and stored at –80 °C.

### Neurochemical DA and DOPAC detection in monkey brains

Dopamine and its metabolite DOPAC tissue content was analyzed as previously described^77^. Samples were weighted and homogenized by sonication in 0.1 N HClO_4_ (1:20, w/v), centrifuged at 10,000xg, the supernatant filtered on micro-centrifuge filters (0.22 µm nylon filter, Costar Spin-X, Corning, NY, USA) and directly injected into the HPLC. The HPLC system was equipped with a Symmetry column (3.0 × 150 mm, C18, 3.5 µm, Waters, Milan, Italy), kept at 38 °C by a Series 1100 thermostat (Agilent Technologies, Waldbronn, Germany). The detector was an ESA Coulochem II (Chelmford, MA, USA), whose analytical cell was set with the first electrode at +200 mV, the second one at −300 mV. Only the second electrode signal was recorded and analyzed. The mobile phase consisted in 80 mM Na_2_HPO_4_, 0.27 mM EDTA, 0.6 mM sodium octyl sulfate, 8% methanol, 4% acetonitrile, pH 2.8 with H_3_PO_4_, delivered at 0.30 ml/min. In these conditions, the detection limit (signal to noise ratio 3:1) was 0.3 pg of DA on column. Data are expressed as pg/mg tissue. Statistical analyses were performed by one-way ANOVA, followed by Fisher’s *post-hoc* comparison, when required.

### Neurochemical analysis of amino acids content

Brain tissue samples of monkeys were homogenized in 1:20 (w/v) 0.2 M TCA, sonicated (3 cycles, 10 s each) and centrifuged at 13,000xg for 20 min. All the precipitated protein pellets from brain samples were stored at −80 °C for protein quantification. Human serum or CSF samples (100 µl) were mixed in a 1:10 dilution with HPLC-grade methanol (900 µl) and centrifuged at 13,000xg for 10 min; supernatants were dried and then suspended in 0.2 M TCA. TCA supernatants from monkey and human samples were then neutralized with NaOH and subjected to pre-column derivatization with o-phthaldialdehyde/N-acetyl-L-cysteine. Diastereoisomer derivatives were resolved on a Simmetry C8 5-μm reversed-phase column (Waters, 4.6 × 250 mm). Identification and quantification were based on retention times and peak areas, compared with those associated with external standards. The identity of peaks was confirmed by selective enzymatic degradation^30,78^. Total protein content of homogenates was determined by Bradford assay method, after resolubilization of the TCA precipitated protein pellets. The detected amino acids concentration in tissue homogenates was normalized by the total protein content and expressed as nmol/mg protein; amino acids level in the serum and CSF was expressed as µM. Statistical analyses in monkey brain were performed by one-way ANOVA, followed by Fisher’s *post-hoc* comparison, when required. Human serum and CSF data were analyzed by Kruskal-Wallis test, followed by Dunn’s test, when required.

### Quantitative Real Time PCR analysis

Total RNAs were extracted using RNeasy® Mini kit (Quiagen, Hilden, Germany), according to the manufacturer’s instructions. Total RNA was purified to eliminate potentially contaminating genomic DNA using recombinant DNAse. We used 0.2 μg of total RNA per sample to synthesize cDNA. After total RNA extraction (0.2 µg), qRT-PCR amplifications were performed with LightCycler 480 SYBR Green I Master (Roche Diagnostic), in a LightCycler 480 Real Time thermocycler (Roche). The following protocol was used: 10 s for initial denaturation at 95 °C followed by 40 cycles consisting of 10 s at 94 °C for denaturation, 10 s at 65 °C for annealing, and 6 s for elongation at 72 °C temperature. The following primers were used for *DDO*, *DAAO* and *SR* cDNA amplification: *DDO* fw: GCAGTGGTTCAGAGAGACCT and *DDO* rev: CGAAATCCCAGAACCACGTC; *DAAO* fw: GGAAGGACACAGTTCTGGGA and *DAAO* rev: CTTCTCTTGCCACCTCCTCA; *SR* fw: AACCAGGTTCCTTTGGTGGA and *SR* rev: CCCTTCAGCTTGGACTGGTA. Transcripts quantities were normalized by the geometric mean of the three housekeeping genes, *Actin b* (*ACTB*), *GAPDH* and Cyclophilin A (*PPIA*), which were amplified using the following primers: *PPIA* fw: TGCTGGACCCAACACAAATG and *PPIA* rev: GTCCACAGTCAGCAATGGTG; *GAPDH* fw: AGGTCGGAGTCAACGGATTT and *GAPDH* rev: ATCTCGCTCCTGGAAGATGG; *ACTB* fw: CTGTGCTATGTCGCCCTAGA and *ACTB* rev: GGAAGGTTGGAAGAGAGCCT. All measurements from each subject were performed in duplicate. mRNA expression was calculated using the relative quantification method (2^−ΔΔCt^). Statistical analyses were performed by one-way ANOVA, followed by Fisher’s *post-hoc* comparison, when required.

### Western blotting

Preparation and immunoblotting were performed as previously described^78^. Frozen, powdered samples from *post-mortem* brains were sonicated in 1% SDS and boiled for 10 min. Proteins were separated by SDS-PAGE and electroblotted onto PVDF membranes (GE-Healthcare). Immunodetections were accomplished by using the following antibodies: anti-SR (1:500, Santa Cruz Biotechnology, Santa Cruz, CA, USA), anti-DAAO (1:1000, Everest Biotech Ltd, Oxfordshire, UK), anti-GluN1 (1:1000, Cell Signaling Technology, Beverly, MA, USA), anti-GluN2A (1:1000, Sigma, St. Louis, MO, USA), anti-GluN2B (1:1000, Cell Signaling Technology), anti-GluR1, anti-GluR2/3 (1:1000, Merck Millipore, Darmstadt, Germany), anti-mGluR2/3 (1:1000, Merck Millipore), anti-mGluR5 (1:1000, Abcam, Cambridge, UK), anti-α-tubulin (1:50000, Sigma), anti-tyrosine hydroxylase (1:2000, Merck Millipore), anti-GAPDH (1:1000, Santa Cruz Biotechnology). Blots were then incubated in horseradish peroxidase-conjugated secondary antibodies. Immunoreactivity was detected by enhanced chemiluminescence (ECL) (GE-Healthcare) and quantified by Quantity One software (Bio-Rad). Optical density values were normalized to α-tubulin or GAPDH for variations in loading and transfer. Statistical analyses were performed by one-way ANOVA, followed by Fisher’s *post-hoc* comparison, when required.

## Supplementary information

Supplementary Information
