# Supplementary material for: The levels of the NMDA receptor co-agonist D-serine are reduced in the substantia nigra of MPTP-lesioned macaques and in the cerebrospinal fluid of Parkinson’s disease patients
Source: Sci Rep. 2019 Jun 20;9:8898. doi: 10.1038/s41598-019-45419-1 (PMC6586824; doi:10.1038/s41598-019-45419-1)

**Supplementary Information**

The levels of the NMDA receptor co-agonist D-serine are reduced in the substantia nigra of MPTP-lesioned macaques and in the cerebrospinal fluid of Parkinson’s disease patients

Tommaso Nuzzo^1,^*, Daniela Punzo^2,3^,*, Paola Devoto^4^, Elena Rosini^5^, Silvia Paciotti^6^, Silvia Sacchi^5^, Qin Li^7,8^, Marie-Laure Thiolat^9,10^, Celine Véga^10^, Massimo Carella^1^, Manolo Carta^4^, Fabrizio Gardoni^11^, Paolo Calabresi^12^, Loredano Pollegioni^5^, Erwan Bezard^7,8,9,10^, Lucilla Parnetti^12^, Francesco Errico^13,@^, Alessandro Usiello^2,3,@^

^1^Translational Neuroscience Unit, IRCCS Casa Sollievo della Sofferenza, 71013 San Giovanni Rotondo, Italy; ^2^Laboratory of Behavioural Neuroscience, Ceinge Biotecnologie Avanzate, 80145 Naples, Italy; ^3^Department of Environmental, Biological and Pharmaceutical Science and Technologies, Università degli Studi della Campania “Luigi Vanvitelli”, 81100 Caserta, Italy; ^4^Department of Biomedical Sciences, University of Cagliari, 09042 Monserrato, Italy; ^5^Department of Biotechnology and Life Sciences, Università degli Studi dell’Insubria, 21100 Varese, Italy; ^6^Department of Pharmaceutical Sciences, University of Perugia, 06122 Perugia, Italy; ^7^Motac Neuroscience, UK-M15 6WE, Manchester, United Kingdom; ^8^Institute of Lab Animal Sciences, China Academy of Medical Sciences, Beijing, China; ^9^Université de Bordeaux, Institut des Maladies Neurodégénératives, Bordeaux, France; ^10^Centre National de la Recherche Scientifique Unité Mixte de Recherche 5293, Institut des Maladies Neurodégénératives, Bordeaux, France; ^11^Dipartimento di Scienze Farmacologiche e Biomolecolari (DiSFeB), Università degli Studi di Milano “La Statale”, 20133 Milan, Italy; ^12^Department of Medicine, Neurology Clinic, University Hospital of Perugia, 06129 Perugia, Italy; ^13^Department of Agricultural Sciences, University of Naples "Federico II", 80055 Portici, Italy.

**Supplementary Methods**

*Materials and enzymes for activity assays*

D-alanine, D-Asp, ATP, L-DOPA, DA, NADH, o-dianisidine (o-DNS), PLP, D-Ser, and L-Ser were purchased from Sigma-Aldrich (St. Luis, MO, USA), D,L-DOPA from Fluorochem (Hadfield, United Kingdom), horseradish peroxidase (HRP) and L-lactate dehydrogenase (LDH) from Roche (Basel, Switzerland). All HPLC solvents were of analytical grade and were used as received.

Recombinant human DAAO (hDAAO) and DAAO from *Rhodotorula gracilis* (RgDAAO) were obtained as previously reported ^1,2^; recombinant hDDO was overexpressed in *E. coli* cells and purified as previously stated ^3^, with minor modifications, and recombinant human SR (hSR) was kindly supplied by Prof. Stefano Bruno (University of Parma, Parma, Italy).

*Assay of oxidative enzymes*

The hDAAO activity was assayed on 28 mM D-alanine as substrate in 75 mM sodium pyrophosphate, pH 8.5 containing 200 μM FAD, at air saturation and 25 °C by measuring the O_2_ consumption with an oxygen electrode ^1^. The hDDO activity was similarly assayed using 15 mM D-Asp in 100 mM sodium pyrophosphate, pH 8.3 containing 40 μM FAD. The same assays were employed to investigate the enzymatic activity of both flavoproteins in the presence of 0.1, 1 or 10 mM (final concentration) D,L-DOPA (in 50 mM potassium phosphate, pH 7.0) or DA, as substrates. The effect of D,L-DOPA and DA (0.1, 1 or 10 mM final concentration) on the enzymatic activity of hDDO on 15 mM D-Asp was also evaluated.

*α,β-Elimination reaction of serine racemase*

The initial rate of L- or D-Ser α,β-elimination by SR was determined by coupling the reaction with pyruvate reduction by lactate dehydrogenase (LDH) and following NADH disappearance at 340 nm ^4^, using a Jasco FP-750 spectrophotometer (Jasco Europe Srl, Cremella, Italy). The assay solution contained 50 mM triethanolamine (TEA), pH 8.0, 150 mM NaCl, 5 mM ditihiothreitol, 50 µM PLP, 2 mM ATP, 2 mM MgCl_2_, 0.3 mM NADH, 30 U/mL L-lactate dehydrogenase, L-Ser (53 and 530 mM) or D-Ser (75 and 750 mM). After incubation at 37 °C for 2 min, the reaction was started by adding 0.5 µM hSR. The activity assays were also measured adding D,L-DOPA, L-DOPA, and DA (0.1, 1 or 10 mM final concentration).

*Racemization reaction of serine racemase*

The initial rate of D-Ser formation from the L-enantiomer by SR was determined by a discontinuous assay based on oxidation of D-Ser by RgDAAO ^5^. The hydrogen peroxide produced by the RgDAAO reaction is reduced by HRP to water and o-DNS is simultaneously oxidized to give a colored compound showing an absorption maximum at 440 nm. The activity assay solution contained 50 mM TEA, pH 8.0, 150 mM NaCl, 50 µM PLP, 2 mM MgCl_2_, 2 mM ATP, 200 mM L-Ser, and 0.5 µM hSR. The reaction mixture (1 ml) was incubated at 37 °C and aliquots were withdrawn at different times (up to 30 min) for D-Ser quantification: 20 µL of reaction mixture were added to the activity assay solution containing 100 mM sodium pyrophosphate, pH 8.5, 1 mM o-DNS, 1 U HRP, and 0.1 U RgDAAO in a 1 ml final volume. The racemization reaction was also carried out in the presence of 0.1, 1 or 10 mM (final concentration) DA.

The racemization reaction of L-DOPA by SR was investigated monitoring the D-DOPA formation by a chiral HPLC analysis. The reaction mixture (1 mL) containing 0.1, 1 or 10 mM L-DOPA (final concentration) was incubated at 37 °C and aliquots were withdrawn at different times for analysis (up to 30 min): 20 µL of reaction mixture was quenched by adding 80 µL of a 1:1 H_2_O/MeOH solution, and centrifuged. The supernatant was analyzed by HPLC using a Jasco apparatus equipped with a UV detector set at 210 nm and fitted with an Astec Chirobiotic TAG column 5 mm (Sigma-Aldrich), length/internal diameter = 250/4.6 mm, eluent H_2_O/MeOH 4:6, flow rate 0.8 mL min^-1^, and column temperature 30 °C. The retention times of L- and D-DOPA were 6.97 and 24.04 min, respectively.

**Supplementary Results**

**Effect of DOPA and dopamine on the activity of human serine racemase, D-aspartate oxidase and D-amino acid oxidase**

The ability of the enantiomeric D- and L-DOPA or of the achiral DA to affect the elimination activity of human SR was evaluated by performing *in vitro* enzymatic assays, using the recombinant purified enzyme on L-Ser, as well as D-Ser, as substrate. The activity of SR was not affected by three different concentrations of both compounds, from 0.1 to 10 mM (the latter too high to be physiologically relevant), and a substrate concentration higher than the Km value (12 and 144 mM for L- and D-Ser, respectively) ^6^ (Supplementary Table S1). Similarly, the racemization of L-Ser by human SR was also unaffected by 0.1 or 1 mM DA. Finally, HPLC analyses demonstrated that human SR does not catalyze the isomerization of DOPA. Indeed, L-DOPA is not converted into the D-enantiomer following incubation with the enzyme. Altogether, we can conclude that the two compounds are neither substrates nor inhibitors of human SR.

Next, we evaluated whether the enzymatic activity of the two amino acid oxidases that overall control the catabolism of D-Ser and D-Asp in humans might be altered by DOPA and DA. As shown in Supplementary Table S2, human DDO is not active on both D- and L-DOPA, and DA (in the 0.1-10 mM concentration range). Furthermore these compounds failed to affect the activity of human DDO on D-Asp, thus demonstrating that they are neither substrates nor inhibitors of this flavoenzyme (Supplementary Table S3). On the other hand, human DAAO is known to efficiently oxidize D-DOPA ^7,8^, with a specific activity 10-fold higher than the value determined on the physiological substrate D-Ser. With D, L-DOPA a substrate inhibition effect at > 15 mM concentration is also apparent. No activity on DA was detected using the human DAAO (Supplementary Table S2).

Overall, these results indicate that the variations in D-amino acids concentrations seen in the monkey brains and CSF of PD patients are not linked to a direct influence of DA and/or L-DOPA upon SR, DAAO and DDO activity since at all doses tested these molecules failed to affect their enzymatic efficiency.

**Supplementary References**

1 Molla, G. *et al.* Characterization of human D-amino acid oxidase. *FEBS Lett* **580**, 2358-2364, doi:10.1016/j.febslet.2006.03.045 (2006).

2 Fantinato, S., Pollegioni, L. & Pilone, M. S. Engineering, expression and purification of a His-tagged chimeric D-amino acid oxidase from Rhodotorula gracilis. *Enzym Microb Technol* **29**, 407-412 (2001).

3 Katane, M. *et al.* Comparative characterization of three D-aspartate oxidases and one D-amino acid oxidase from Caenorhabditis elegans. *Chem Biodivers* **7**, 1424-1434, doi:10.1002/cbdv.200900294 (2010).

4 Marchetti, M. *et al.* Regulation of human serine racemase activity and dynamics by halides, ATP and malonate. *Amino Acids* **47**, 163-173, doi:10.1007/s00726-014-1856-2 (2015).

5 Rosini, E., Caldinelli, L. & Piubelli, L. Assays of D-Amino Acid Oxidase Activity. *Front Mol Biosci* **4**, 102, doi:10.3389/fmolb.2017.00102 (2017).

6 Marchetti, M. *et al.* ATP binding to human serine racemase is cooperative and modulated by glycine. *FEBS J* **280**, 5853-5863, doi:10.1111/febs.12510 (2013).

7 Murtas, G., Sacchi, S., Valentino, M. & Pollegioni, L. Biochemical Properties of Human D-Amino Acid Oxidase. *Front Mol Biosci* **4**, 88, doi:10.3389/fmolb.2017.00088 (2017).

8 Wu, M., Zhou, X. J., Konno, R. & Wang, Y. X. D-dopa is unidirectionally converted to L-dopa by D-amino acid oxidase, followed by dopa transaminase. *Clin Exp Pharmacol Physiol* **33**, 1042-1046, doi:10.1111/j.1440-1681.2006.04484.x (2006).

**Supplementary Table S1.** Effect of different concentrations of D,L-DOPA or dopamine on the elimination activity of human SR using 530 mM L-serine (53 mM for values in brackets) or 750 mM D-serine (75 mM for values in brackets) as substrate.

|  |  | **Residual activity (%)** | |
| --- | --- | --- | --- |
| *[D,L-DOPA] (mM)* |  | *L-Ser* | *D-Ser* |
| 0.1 |  | 106 ± 5.6 (88.8 ± 6.5) | 96.7 ± 4.6 (89.6 ± 0.9) |
| 1 |  | 103 ± 5.5 (87.4 ± 6.0) | 95.0 ± 3.6 (89.6 ± 1.2) |
| 10 |  | 103 ± 5.8 (76.9 ± 6.1) | 80.0 ± 16.9 (75.9 ± 6.6) |
| *[Dopamine] (mM)* |  |  |  |
| 0.1 |  | 109 ± 5.9 (106 ± 7.1) | 96.7 ± 4.6 (96.5 ± 9.6) |
| 1 |  | 102 ± 5.7 (99.3 ± 6.8) | 83.3 ± 3.2 (96.5 ± 7.5) |
| 10 |  | 114 ± 8.2 (94.2 ± 14.8) | 86.7 ± 7.3 (93.1 ± 18.4) |

**Supplementary Table S2.** Activity of human DDO (hDDO) and DAAO (hDAAO) on D,L-DOPA and dopamine (at 0.1, 1 and 10 mM final concentration). * 15 mM D-aspartate and 28 mM D-alanine for hDDO and hDAAO, respectively; ** no activity even using a 10-fold higher amount of enzyme than for the standard assay; *** a substrate inhibition effect is apparent at concentration > 15 mM.

|  |  | **Activity (U/mg)** | | |
| --- | --- | --- | --- | --- |
| *Enzyme* |  | *Reference substrate** | *D,L-DOPA* | *Dopamine* |
| hDDO |  | 94.9 ± 3.1 | no activity** | no activity** |
| hDAAO |  | 7.1 ± 0.1 | 60.5 ± 7.4*** | no activity** |

**Supplementary Table S3.** Effect of different concentrations of D,L-DOPA or dopamine on the oxidative deamination activity of human DDO on 15 mM D-aspartate as substrate.

|  |  | **Residual activity (%)** | |
| --- | --- | --- | --- |
| *[D,L-DOPA] (mM)* |  |  |  |
| 0.1 |  | 95.3 ± 4.7 |  |
| 1 |  | 98.1 ± 2.8 |  |
| 10 |  | 86.9 ± 2.8 |  |
| *[Dopamine] (mM)* |  |  |  |
| 0.1 |  | 98.5 ± 2.4 |  |
| 1 |  | 88.9 ± 6.2 |  |
| 10 |  | 78.1 ± 11.3 |  |

**Supplementary Figure S1.** Full-length blots. Abbreviations: SN = Substantia nigra; Pu = Putamen; C = Control; M = MPTP; M+LD = MPTP + L-DOPA.

Raw blots of figure 4j,k.


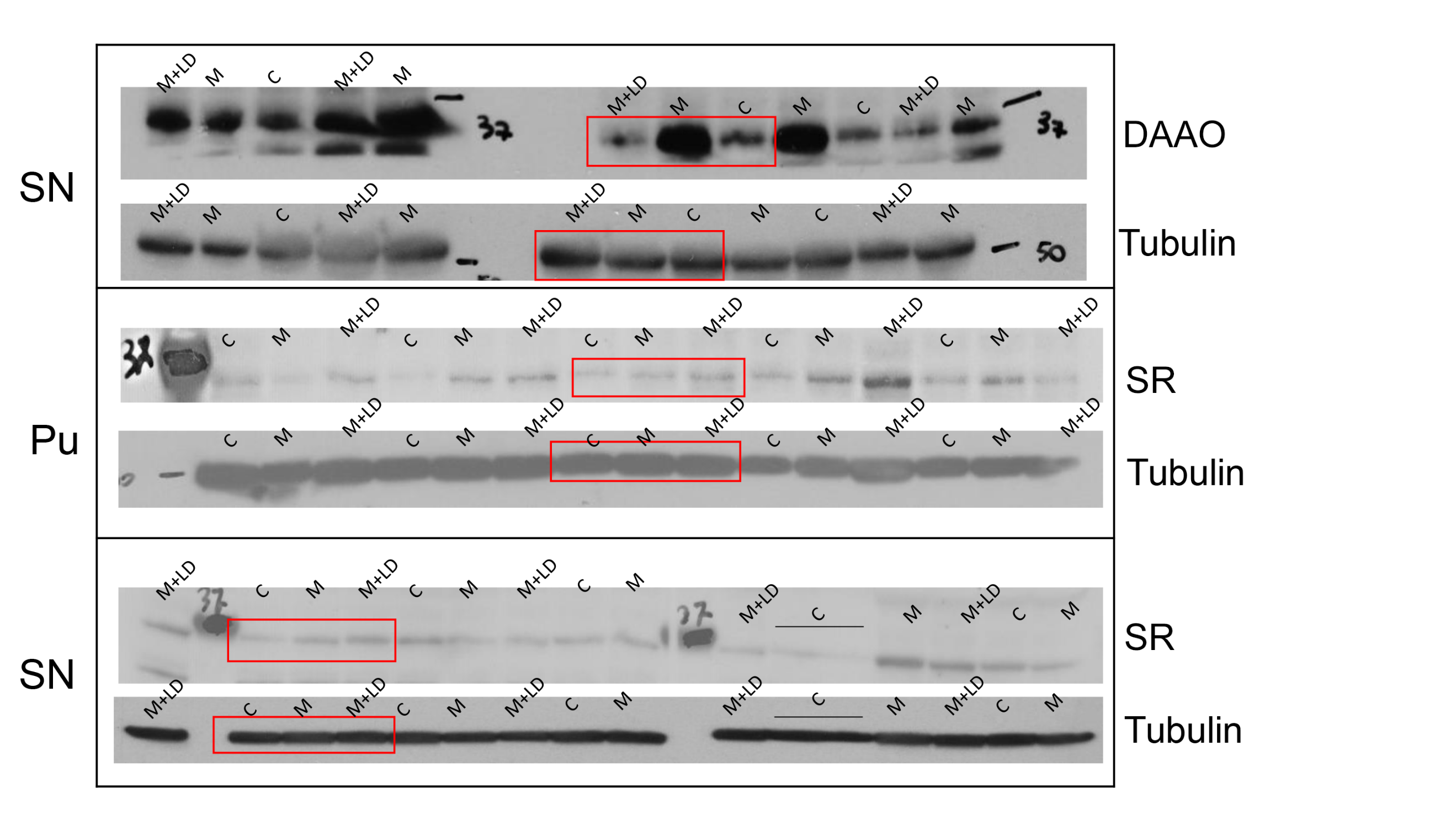


Raw blots of figure 5 (Putamen)


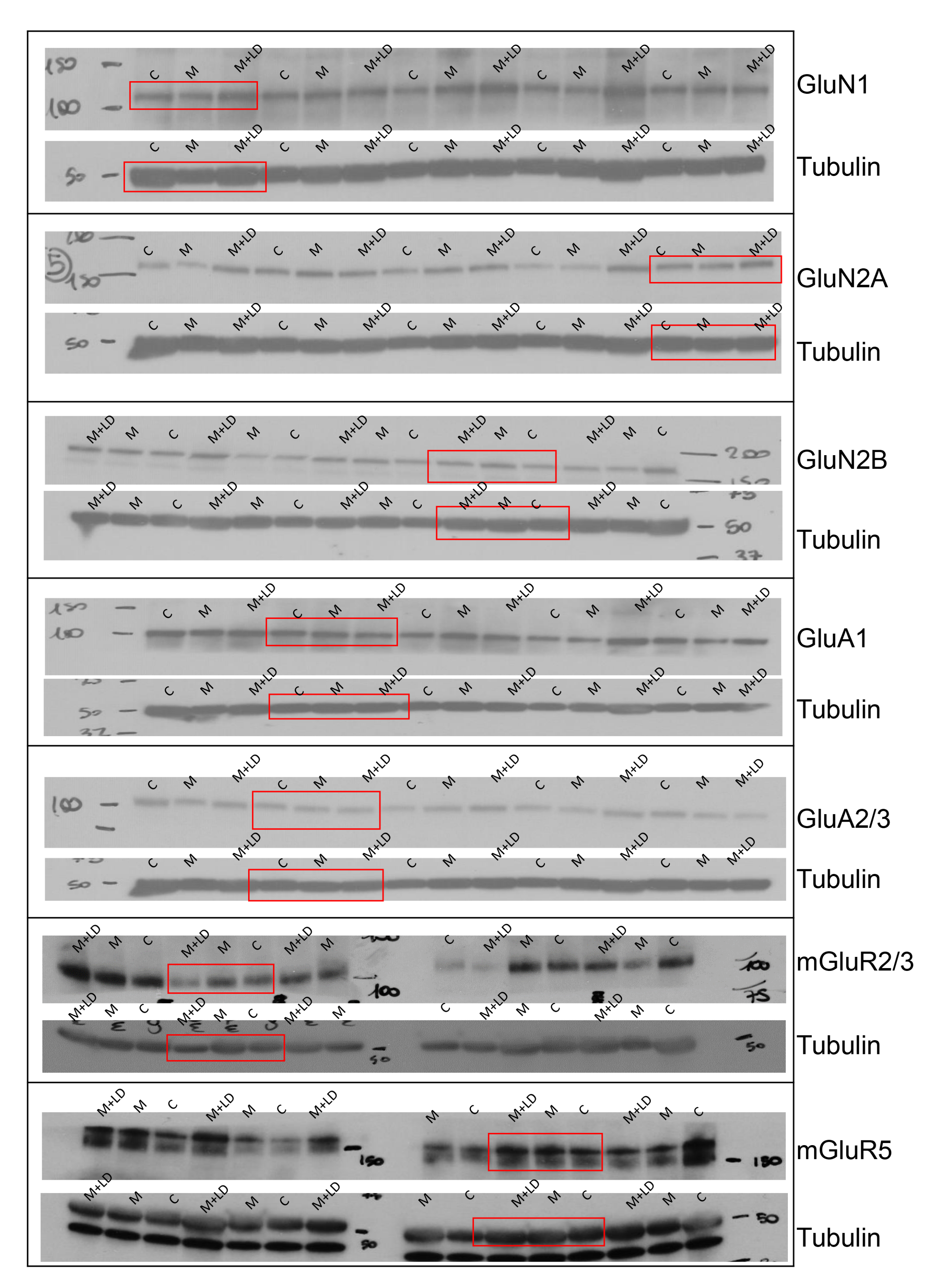


Raw blots of figure 5 (*Substantia nigra*)


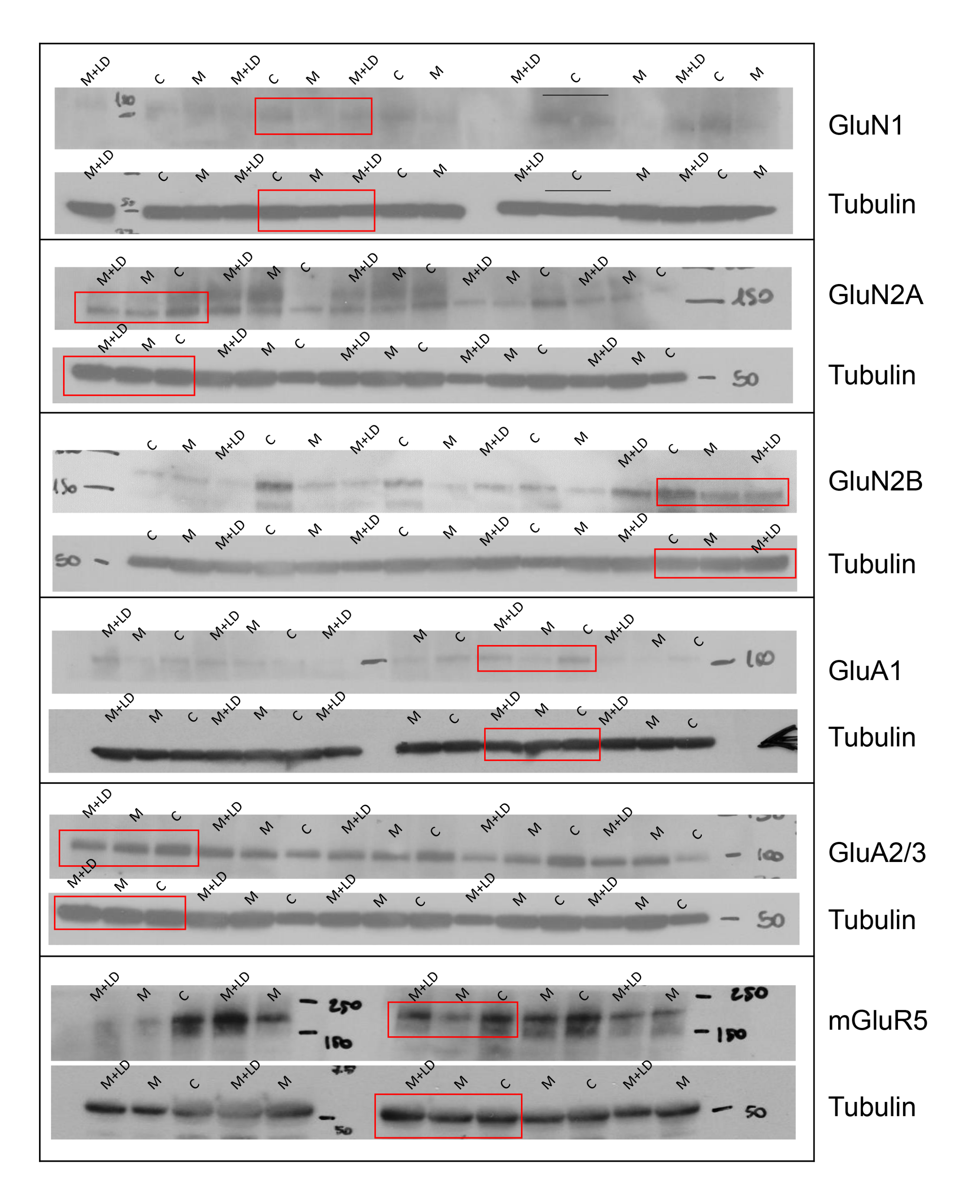

Supplement: Supplementary file 1 — Supplementary Information [file 41598_2019_45419_MOESM1_ESM.docx]
